# Connexin 43 Upregulation in Mouse Lungs during Ovalbumin-Induced Asthma

**DOI:** 10.1371/journal.pone.0144106

**Published:** 2015-12-02

**Authors:** Yin Yao, Qing-Xiang Zeng, Xue-Quan Deng, Guan-Nan Tang, Jie-Bo Guo, Yue-Qi Sun, Kun Ru, Alicia N. Rizzo, Jian-Bo Shi, Qing-Ling Fu

**Affiliations:** 1 Otorhinolaryngology Hospital, The First Affiliated Hospital, Sun Yat-sen University, Guangzhou, Guangdong province, China; 2 Otorhinolaryngology Institute, Sun Yat-sen University, Guangzhou, Guangdong province, China; 3 Division of Pulmonary, Critical Care, Sleep, and Allergy, University of Illinois at Chicago, Chicago, Illinois, United States of America; University of Southampton, UNITED KINGDOM

## Abstract

**Background:**

Connexin (Cx)-based gap junction channels play important roles in the inflammatory response. Cx43 is involved in the pathogenesis of some lung diseases such as acute lung injury. However, the Cx43 expression in asthma is unclear. In the present study, we used a murine model of ovalbumin (OVA)-induced allergic airway disease to examine the levels of Cx43 and analyze the relationship between Cx43 and airway inflammation in allergic airway disease.

**Methods:**

Asthma was induced in mice via sensitization and challenge with OVA. Cx43 mRNA and protein expression levels were investigated via QT-PCR, western blot, and immunohistochemistry 0 h, 8 h, 1 d, 2 d and 4 d after the first challenge. The relationship between Cx43 protein levels and inflammatory cell infiltration, cytokine levels was analyzed.

**Results:**

The OVA-induced mice exhibited typical pathological features of asthma, including airway hyper-responsiveness; strong inflammatory cell infiltration surrounding the bronchia and vessels; many inflammatory cells in the bronchoalveolar lavage fluid (BALF); higher IL-4, IL-5 and IL-13 levels; and high OVA specific IgE levels. Low Cx43 expression was detected in the lungs of control (PBS) mice. A dramatic increase in the Cx43 mRNA and protein levels was found in the asthmatic mice. Cx43 mRNA and protein expression levels increased in a time-dependent manner in asthma mice, and Cx43 was mostly localized in the alveolar and bronchial epithelial layers. Moreover, lung Cx43 protein levels showed a significant positive correlation with inflammatory cell infiltration in the airway and IL-4 and IL-5 levels in the BALF at different time points after challenge. Interestingly, the increase in Cx43 mRNA and protein levels occurred prior to the appearance of the inflammatory cell infiltration.

**Conclusion:**

Our data suggest that there is a strong upregulation of Cx43 mRNA and protein levels in the lungs in asthma. Cx43 levels also exhibited a positive correlation with allergic airway inflammation. Cx43 may represent a target to treat allergic airway diseases in the future.

## Introduction

Asthma is a common heterogeneous respiratory disease that has increased over the past 50 years [1]. Asthma is caused by allergen inhalation, leading to airway hyper-responsiveness, inflammation infiltration, mucous production, structural changes in the airway walls and airway obstruction [2]. Recently, a substantial body of evidence suggested the importance of epithelial cells [3,4] and innate lymphoid cells [5,6] in initiating and sustaining the allergic cascade. Despite increasing evidence regarding the import role of epithelial cells in airway allergy inflammation, including contribution of activation and survival of mast cells, basophil and eosinophils [7], the underlying molecular mechanisms remain incompletely characterized, especially regarding the cell-cell interactions and the molecules involved.

Direct cell-to-cell communication mediated by gap junction channels (GJCs) plays several central roles in cell growth and differentiation, cell cycle regulation and carcinogenesis [8–10]. GJCs are essential for coordinating tissue homeostasis and regulating inflammatory responses, which directly link the cytoplasm, which allows for conduction of intercellular signals between adjacent cells. This behavior enables the lung, which is composed of many types of cells, to behave as an integrated system [11–13]. Connexins from both cells dock to form a GJC and allow the passage of metabolites (i.e., ATP), second messengers and small soluble molecules between the cell interior and the interstitial space, thereby coupling the cells both electrically and metabolically [14,15]. More than twenty types of connexins have been characterized in humans, including Cx26, Cx32, Cx37, Cx40, Cx43 and Cx46. Cx43 is one of the most frequently expressed connexins [11,12].

As an inflammatory mediator and signal conductor, Cx43 plays an important role in the pathogenesis of many lung diseases, including acute lung injury (ALI), cystic fibrosis (CF), pulmonary arterial hypertension (PAH) and cancer [11]. Cx43 expression is increased at the alveolar level in ALI [16] and in the pulmonary arterial wall during PAH [17]. The pro-inflammatory role of Cx43 was also confirmed by lipopolysaccharide (LPS)-mediated inflammation using Cx43+/- mice [18]. Recently, a study showed that OVA challenged mice developed typical asthma, accompanying decrease of connexin37, another family member of connexins [19]. These studies lead us to investigate the importance of Cx43 in asthma-mediated airway abnormality [20].

In the present study, we used a murine ovalbumin (OVA)-induced asthma model to examine the expression and location of Cx43 during allergic disease and the correlation between Cx43 expression and airway allergic inflammation.

## Materials and Methods

### Animals and experimental protocol

Female BALB/c mice (4- to 6-wk-old) were purchased from the Guangdong Medical Laboratory Animal Center (Guangzhou, China) and acclimatized for 1 week prior to starting the experiments. The animals were maintained under specific pathogen-free conditions and fed by professional technicians in The Animal Center, Sun Yat-sen University. All procedures were approved by the Institutional Animal Care and Use Committee, Sun Yat-sen University (No. IACUC 20110228002).

**Fig 1:**
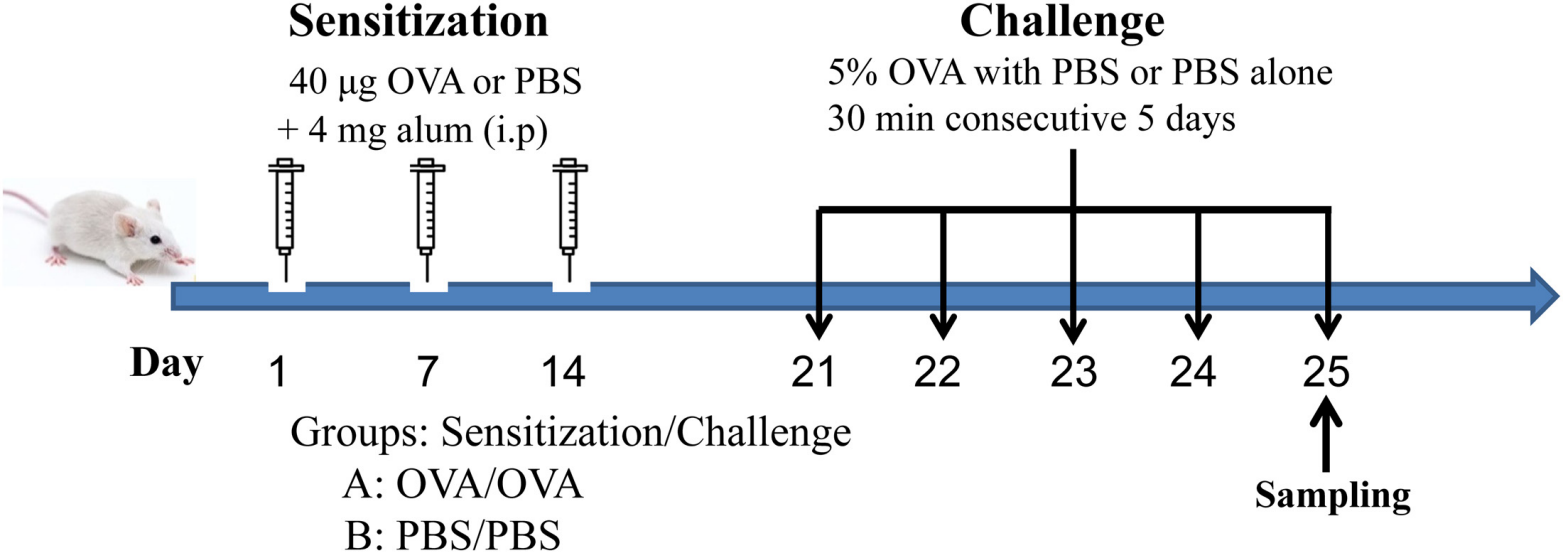
A mouse model of OVA-induced asthma. For sensitization, BALB/c mice received an i.p injection of OVA or PBS with aluminum hydroxide on days 1, 7 and 14. From days 21 to 25, the mice were challenged with aerosolized 5% OVA or PBS in a plexiglass chamber for 30 minutes per day. Samples were collected on day 25. PBS, phosphate-buffered saline; i.p, intraperitoneal; OVA, ovalbumin.

OVA—induced asthma was performed as described in our previous report with a minor modification (Fig 1) [20]. Briefly, the mice were sensitized with an i.p. injection of 40 μg/mouse of OVA (grade V, Sigma, St. Louis, MO, USA) and 4 mg of aluminum hydroxide (Thermo Scientific, Rockford, MD, USA) in 100 μl of pyrogen-free phosphate-buffered saline (PBS, PH = 7.3) or equal amounts of PBS on days 1, 7 and 14. Then, the mice were challenged with 5% aerosolized OVA or PBS through an air-compressing nebulizer in a plexiglass chamber (403A, Yuyue, Danyang, Jiangsu, China) for 30 min for five successive days (days 21–25). Mice were divided into two groups: (A) PBS/PBS mice that were sensitized and challenged with PBS; (B) OVA/OVA mice that were sensitized and challenged with OVA. Mice were sacrificed on day 26 for airway hyper-reactivity (AHR) measurements and sacrificed on day 25 (4 days after the first challenge) for the examination of inflammatory infiltrates, cytokine production, serum Ig proteins and Cx43 expression. To further examine Cx43 expression and its relationship with inflammatory infiltrates and cytokine production, mice were sacrificed at different time points (0 h, 1 h, 8 h, 1 d and 2 d) after the first challenge and samples were collected to evaluate the inflammatory infiltrates, cytokine levels, serum IgE levels and Cx43 expression.

All efforts were made to minimize the number of animals used and their suffering. During the experiments, the animals were anesthetized by intraperitoneal administration of a mixture of pentobarbital sodium (45 mg/mL, Sigma-Aldrich, St. Louis, MO, USA). We determined the degree of anesthesia by toe clip and corneal reflections. The mice were sacrificed by intraperitoneal injection with an overdose of anesthetic after collecting blood via orbital puncture or AHR measurements.

These studies were repeated 3 times. Four animals for each group were used for the first time and all of the parameters mentioned in this study except the cytokines were measured. Six animals for each group were used for the second and third times and were detected all of the parameters and did the correlations between Cx43 proteins and airway allergic inflammation. Similar results were attained from the three experiments.

### Airway hyper-reactivity measurements

AHR to methacholine was measured using a single-chamber, whole body plethysmograph (WBP system, Buxco Electronics, USA) on day 26 as previously described [21]. First, unrestrained and spontaneously breathing mice were placed in an acclimatization chamber. A baseline response was determined, and then the mice were given nebulized PBS with increasing methacholine concentrations (6.25, 12.5, 25, 50 and 100 mg/ml) (Sigma-Aldrich, St. Louis, MO, USA). The reading interval was set to 5 minutes after nebulization. Enhanced pause (Penh) values for each methacholine dosage were determined using the following equation: [Penh = pause6 (peak inspiratory box flow/peak expiratory box flow)]. The data are shown as Pech%, which represents the percent changes of Pech from the corresponding baseline values. Penh values equal to 100% and 200% of the baseline (PC100 and PC200) were obtained by linear interpolation of the concentration response curves between the final two doses of the respective stimulator.

### Histology analysis and immunohistochemistry

Harvested lungs were fixed in 4% formalin overnight and embedded in paraffin for H&E, periodic acid—Schiff (PAS), or Masson’s staining or embedded in optimum cutting temperature compound (OCT) for immunohistochemistry. Inflammation scores (inflammation around the bronchial, vascular and total tissues) in the lungs were determined in a blinded fashion using a reproducible scoring system described in our previous report [20]. For quantifying the goblet cell hyperplasia, the percentage of PAS-positive cells in epithelial areas was examined from 8 to 10 tissue sections per mouse [20]. For immunohistochemical staining [16], non-specific binding was blocked using 10% goat serum in PBS for 1 h, and the samples were incubated with primary mouse Abs to Cx43 (1:500, BD Biosciences, NJ, USA) overnight at 4°C. After washing, the slides were incubated with Alexa Fluor 568 goat anti-mouse secondary antibody at room temperature for 2 h. The nuclei were counterstained using DAPI (Sigma-Aldrich, MO, USA). Isotype-matched negative control Abs (IgG1, R&D Systems, Minneapolis, MN, USA) were used on the same lung tissues. Tissues were also incubated only with secondary antibody but not primary antibody as the negative control.

### Cytokine and OVA-specific Ig protein measurements

Bronchoalveolar lavage fluid (BALF) and serum were harvested as described in our previous report [20]. The IL-4, IL-5, IL-13 and interferon (IFN)-γ levels in the BALF supernatants were measured using sandwich enzyme-linked immunosorbent assays (ELISA) following the manufacturer’s instructions (R&D Systems, Minneapolis, MN, USA).

OVA-specific IgE, IgG1 and IgG2a were measured as follows. Briefly, 96-well plates were coated with 10 g/ml OVA overnight at 4°C in PBS (pH = 9.6), and then blocked for 1 h at 4°C with 1% BSA (Amersham Biosciences, Little Chalfont Buckinghamshire, UK) in PBS. The plates were washed and 50 μl of the diluted serum sample (1:100,000 for IgG1, 1:200 for IgG2a and no dilution for IgE) was applied to the wells in triplicate and incubated for 2 h at room temperature. After washing, the respective secondary Abs were added to the IgG1, IgG2a or IgE wells (BD Biosciences, NJ, USA) at dilutions of 1:2,000 in PBS and incubated at room temperature for 2 h. After washing, the plates were incubated with 100 μl of TMB substrate (Cell Signaling, MA, USA) at room temperature for another 30 min. Then, 50 μl of stop solution was added to each well. Absorption was measured at 450 nm with a PowerWaveX microplate absorbance reader (Bio-Tek Instruments, VT, USA). Data are presented as OD values from identical dilutions in the linear range of the readings.

### Western blot analysis

To measure the Cx43 levels, the animals were sacrificed 0 h, 1 h, 8 h, 1 d, 2 d and 4 d after the first challenge. The lungs were dissected and homogenized in lysis buffer (10 mM Tris, pH 7.4, 150 mM NaCl, 1 mM EDTA, and 1 mM EGTA) supplemented with 1% protease inhibitor and 1% phosphatase inhibitor cocktails (Sigma, MO, USA). After centrifugation at 12,000 x g for 30 minutes, the protein concentration of the supernatants was measured using a bicinchoninic acid (BCA) protein assay kit (Bio-Rad Laboratories, Hercules, CA, USA). An 80 μg aliquot of proteins from each sample was subjected to 10% SDS-PAGE and transferred onto a PVDF membrane. The membranes were blocked with 5% nonfat dry milk in Tris-buffered saline containing 0.1% Tween 20 (TBST) for 1 h at room temperature. Incubation with a primary mouse Ab to Cx43 (1:1000, Millipore, CA, USA) was performed for 16 h at 4°C. After washing, the membranes were incubated with a horseradish peroxidase-conjugated secondary antibody (1:2,000, Jackson ImmunoResearch Inc., PA, USA) in 5% nonfat milk in TBST for 1 h at room temperature, and the immunoreactive proteins were detected using the enhanced chemiluminescence method (ECL; Millipore, MA, USA) and scanned using a G:BOX system (Syngene, MD, USA). Protein loading was controlled using a monoclonal mouse antibody against anti-GAPDH (1:2,000, Abcam, Cambridge, UK). The intensity of each band was quantified with ImageJ (National Institutes of Health, MD, USA). All western blot experiments were repeated three times. Protein levels were expressed as relative values compared with the lungs at 0 h after the first challenge in the PBS/PBS group.

### Quantitative real-time PCR

We isolated lungs at different time points as described above, mixed the pellets with 10 volumes of TRIzol reagent (Invitrogen, Paisley, UK), and then stored them at -80°C prior to use. Total RNA was extracted and cDNA was synthesized using an M-MLV Reverse Transcriptase Kit (Promega, WI, USA). cDNA equivalent to 1 ng of total RNA was used to perform quantitative PCR with a 9700 PCR System (ABI, GeneAmp, USA). Additionally, the SYBR Green Master Mix was also utilized (Roche, USA). Relative gene expression was calculated using the comparative CT method. To eliminate the data bias from single inner control, 18S-rRNA, GAPDH and β-actin were used as the housekeeping genes for normalization. The geometric mean of relative expression normalized to each inner control was used as the transcript level of target genes. Finally, mRNA levels were expressed as relative values compared with the levels in the PBS/PBS group lungs at 0 h after the first challenge. The following PCR primers were used to amplify specific portions of the cDNA products: mouse Cx43, 5’-GAACACGGCAAGGTGAAGAT-3’ and 5’- GAGCGAGAGACACCAAGGAC-3’; mouse 18S Ribosomal RNA (18S-rRNA), 5’- AGGATGTGAAGGATGGGAAG -3’ and 5’- TTCTTCAGCCTCTCCAGGTC -3’; mouse GAPDH, 5’- CGACTTCAACAGCAACTCCCACTCTTCC-3’ and 5’- TGGGTGGTCCAGGGTTTCTTACTCCTT-3’; mouse β-actin, forward 5’-ATGGATGACGATATCGCTGCGC-3’ and 5’-GCAGCACAGGGTGCTCCTCA-3’.

### Statistical Analysis

The experimental data were expressed as the mean ± SEM and analyzed using Student’s t-test or Mann-Whitney-U test for two groups or one-way analysis of variance (ANOVA) followed by a post-hoc test (Dunnett’s T3) or Tukey test for comparisons of more than two groups. The Pearson product-moment correlation coefficient (r) was used to assess the relationship between Cx43 levels and inflammatory infiltrates. Statistical analyses were performed using the SPSS 19.0 software, and p < 0.05 was considered statistically significant.

## Results

### Systematic administration of OVA induced AHR and inflammatory infiltrate in the lung

**Fig 2:**
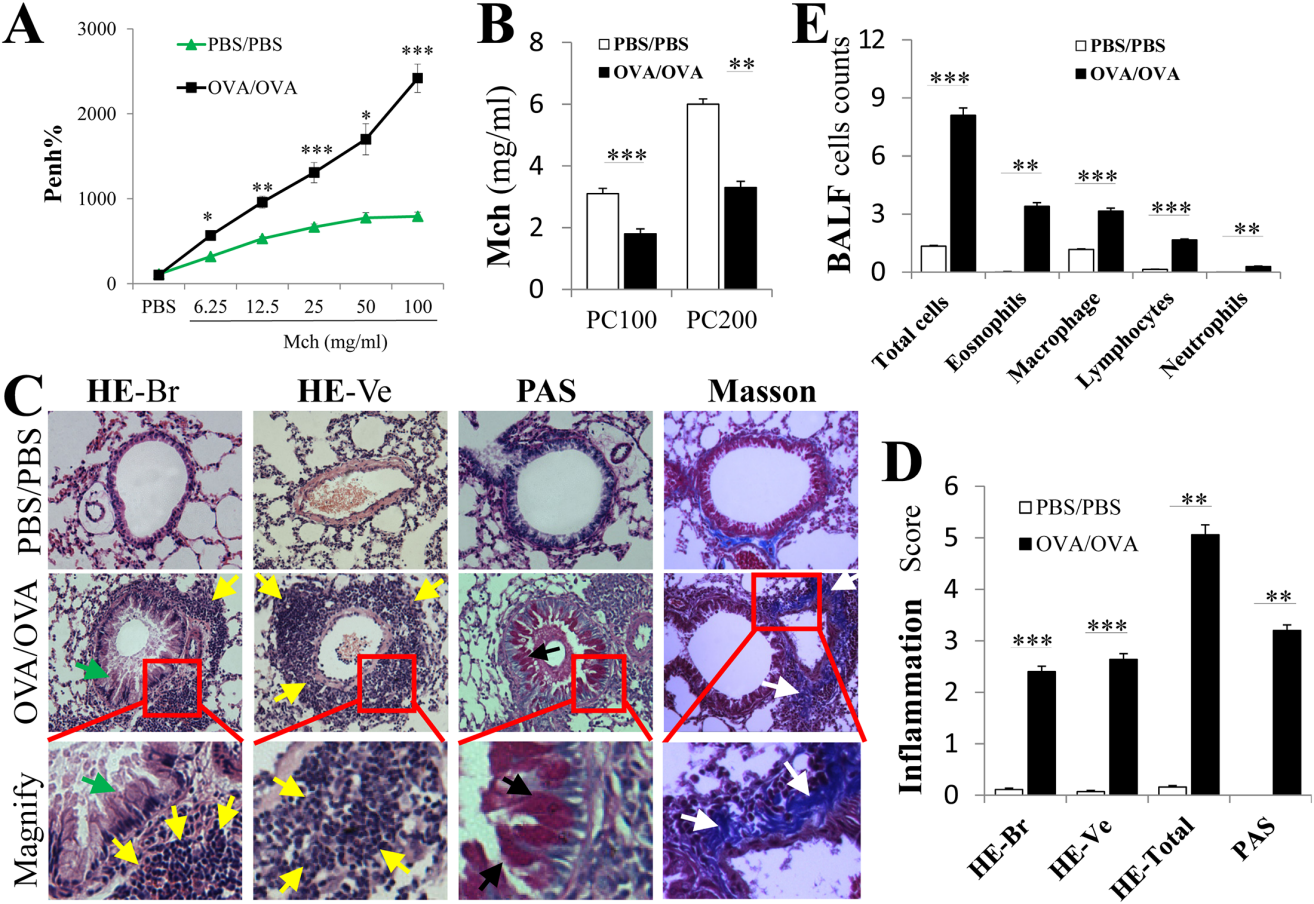
Airway hyper-reactivity determination and airway inflammation in the lung of asthma mice. The animals were sacrificed on day 25 to determine tissue inflammation and on day 26 for airway hyper-reactivity (AHR). (A) The OVA/OVA mice presented a remarkably high airway responsiveness to increasing metacholine (Mch) doses (6.25, 12.5, 25, 50 and 100 mg/ml). *, P < .05; ***, P < .001 compared with the control group at the same Mch concentration. (B) PC100 and PC200 were obtained by linear interpolation of the concentration response curve between the final two doses of the respective provocative agent. (C) Representative H&E, PAS and Masson’s stained lung section photomicrographs are shown for each group. The OVA/OVA mice showed apparent inflammatory infiltrates (yellow arrows), including peribronchial (HE-Br) and perivessel (HE-Ve) inflammation, swollen bronchial epithelial cells with increased mucus (green arrows), numerous mucus-containing cells (PAS, black arrows) and collagen deposition (white arrows, blue staining for Masson’s) compared with the PBS/PBS mice. The original magnification was ×200. (D) The airway inflammation scores were based on the HE and PAS staining. The HE total represented the scores from HE stains from both peribronchial and perivessel cells. (E) Cell counts were obtained from the total inflammatory cells, eosinophils, macrophages, lymphocytes and neutrophils in the BALF. Mean ± SEM. *, P < .05; **, P < .01; ***, P < .001 compared with the PBS/PBS group. AHR, airway hyper-reactivity. BALF, bronchoalveolar lavage fluid; H&E, hematoxylin and eosin; HE(Br), HE stain peribronchial; HE(Ve), HE stain perivessel; OVA, ovalbumin; PAS, periodic acid—Schiff; PBS, phosphate-buffered saline; Pech%, the percent changes of Pech from corresponding baseline values; PC100, increased Penh to 100% of baseline; PC200, increased Penh to 200% of baseline. N = 6 mice for each group. Results are representative of three independent experiments.

As shown in Fig 2A, different dosages of methacholine (Mch) did not cause a significant difference of Penh values in the PBS/PBS groups. However, Mch caused a dose-dependent Penh% increase in OVA/OVA mice. Meanwhile, PC100 and PC200 which represent airway sensitivity to Mch were significant decreased in OVA/OVA mice comparing to PBS/PBS mice (Fig 2B, P < 0.001).

To assess airway inflammation, goblet cell metaplasia and subepithelial fibrosis levels, the mouse lungs were performed H&E, PAS and Masson’s staining, respectively (Fig 2C). HE-Br staining indicated that OVA resulted in inflammatory responses, including edema and inflammatory cell infiltration (Fig 2C HE-Br). HE-Ve staining also indicated that there was inflammatory response to OVA-induction around lung blood vessels (Fig 2C HE-Ve). PAS staining revealed swollen bronchial epithelial cells and higher mucus secretion in OVA/OVA mice bronchi (Fig 2C PAS). Masson staining showed that collagen deposition was observed around bronchi in OVA/OVA mouse lung (Fig 2C Masson). Comparing to PBS/PBS mice, significantly higher airway inflammation scores for H&E and PAS-staining and higher combined scores in the areas surrounding the bronchi and vasculature were observed in OVA/OVA mice (Fig 2D, P < 0.01 or 0.001). Finally, compared to PBS/PBS mice, OVA sensitization and challenge induced a significant increase for total cells as well as eosinophils, macrophages, lymphocytes and neutrophils in BALF (Fig 2E, P < 0.01 or 0.001).

### OVA induced a Th2 cytokine response in the BALF and high Ig protein levels in the serum

**Fig 3:**
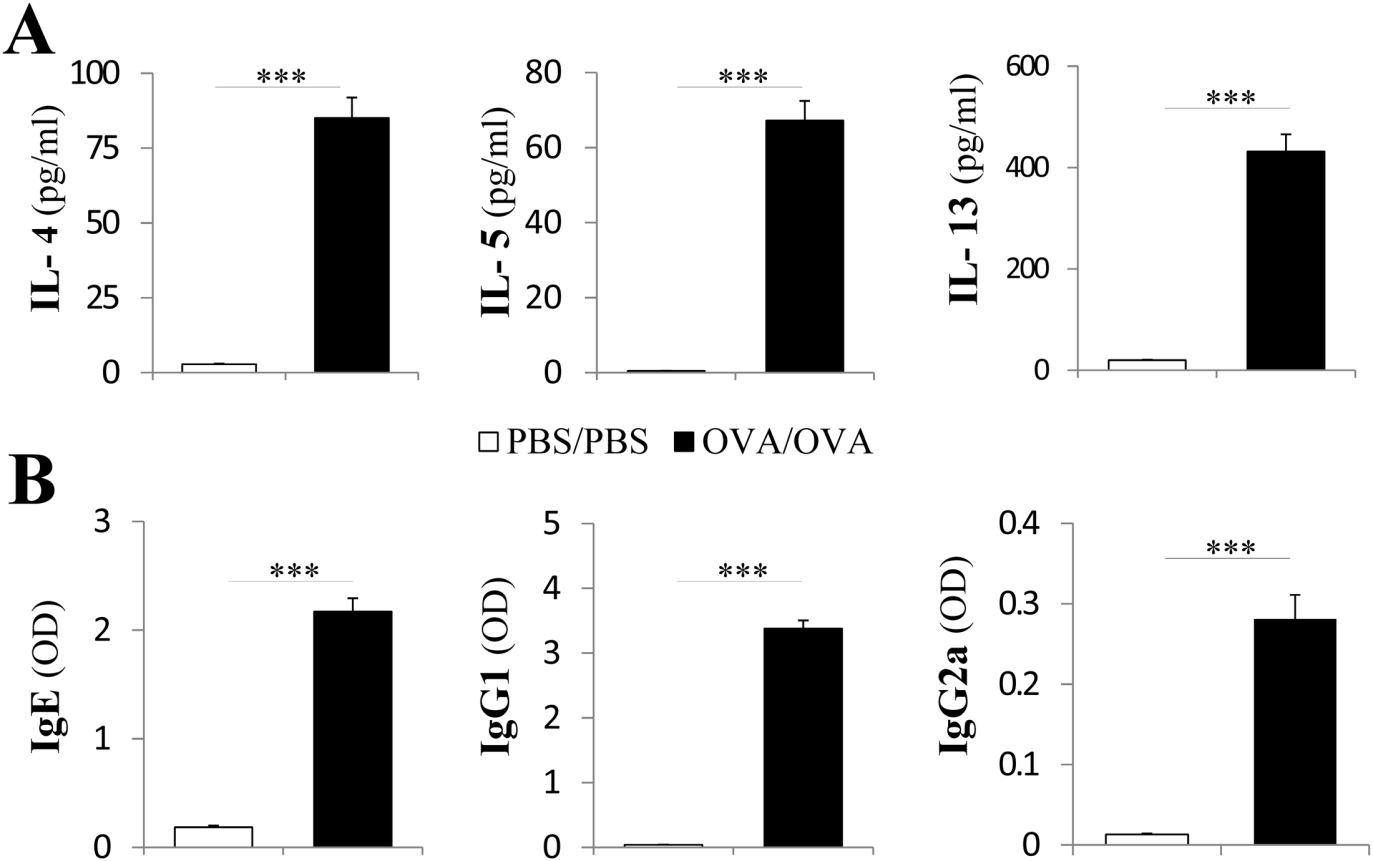
OVA induced increased BALF Th2 cytokine levels and serum Ig protein levels. (A) BALF IL-4, IL-5 and IL-13 levels. (B) Serum IgE, IgG1 and IgG2a levels. Mean ± SEM. *, P < .05; **, P < .01; ***, P < .001. N = 6 in each group. Results are representative of three independent experiments.

The abnormal Th2 response is the key feature of asthma [2]. BALF cytokine assay showed that the IL-4, IL-5 and IL-13 concentration in BALF were significantly higher in the OVA/OVA mice compared with the PBS/PBS mice (Fig 3A, P < 0.001). Moreover, the OVA-specific Igs, including IgE, IgG1 and IgG2a, were markedly increased in the OVA/OVA mice compared to PBS/PBS mice (Fig 3B, P < 0.001).

### Characterization of OVA-mediated Cx43 induction in mouse lung

**Fig 4:**
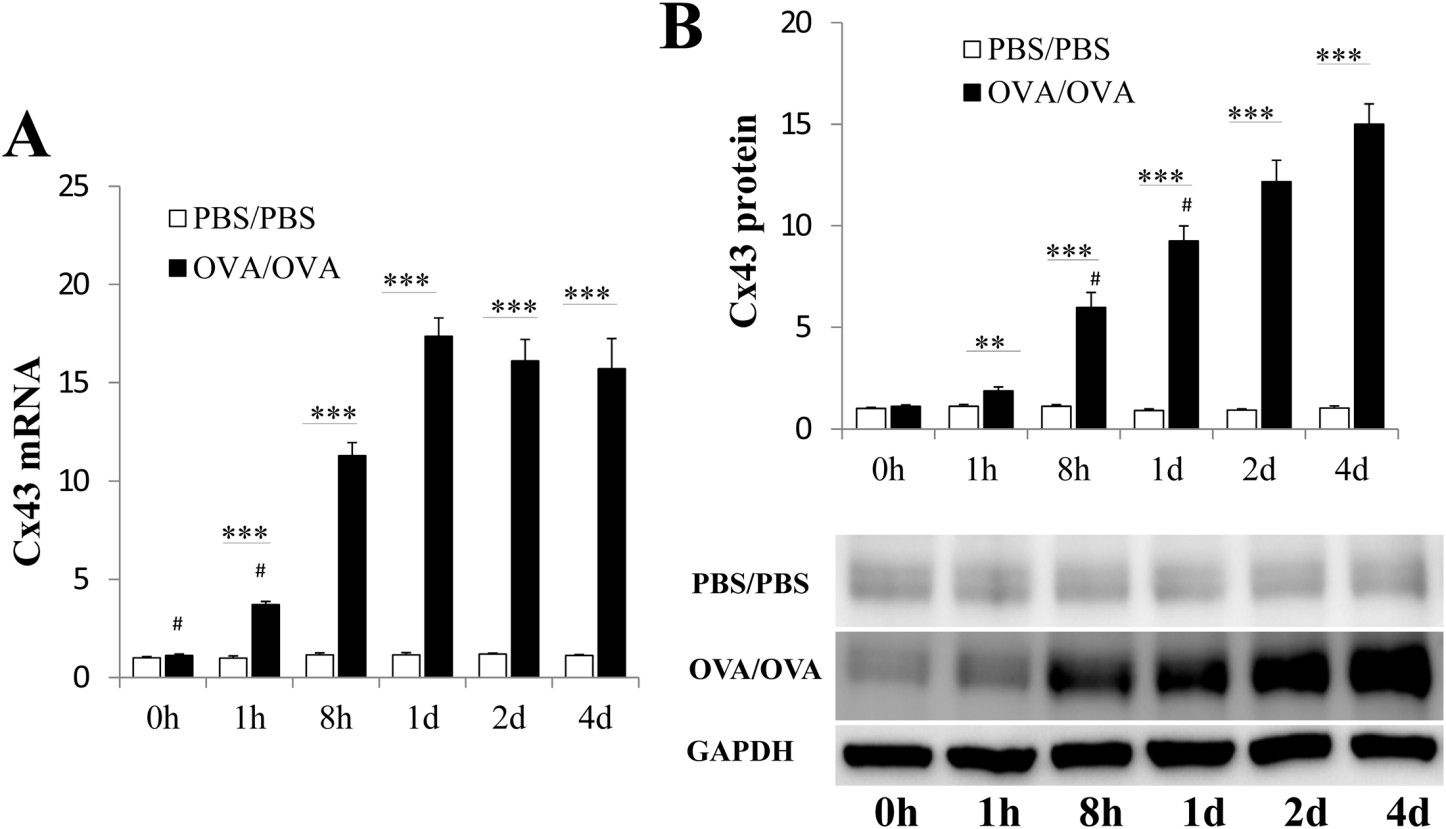
Cx43 mRNA and protein levels in the lung tissue in asthma mice. Samples were collected at 0 h, 1 h, 8 h, 1 d, 2 d and 4 d after the first challenge. (A) Quantitative analysis of lung Cx43 mRNA expression using real-time RT-PCR in the OVA/OVA and PBS/PBS mice. (B) Western blot analyses of Cx43 protein levels at different time points after the first challenge in the OVA/OVA and PBS/PBS groups. The Cx43 protein levels increased in a time-dependent manner in the OVA/OVA group. The data are represented as the means ± SEMs from 6 mice per group. Results are representative of three independent experiments. #, P < .05, .01 or .001 compared with the following time points. *, P < .05; **, P < .01; ***, P < .001 compared with the PBS/PBS group. The Cx43 mRNA and protein levels were relative to the PBS/PBS group 0 h after challenge.

We next investigated Cx43 mRNA and protein expression levels in mouse lungs. QT-PCR analysis revealed that Cx43 mRNA expression gradually increased and reached a plateau of approximately 14-fold 1 d after the first challenge (Fig 4A, P < 0.05, 0.01 or 0.001 at 1, 2, and 4 d compared with 0 h, 1 h and 8 h, respectively). No significant Cx43 mRNA expression changes were observed in the PBS/PBS group in different time points. Cx43 mRNA expression was higher in the OVA/OVA group compared with the PBS/PBS group at the 1 h, 8 h, 1 d, 2 d and 4 d time points after the first challenge (all P < 0.001).

Western blot analysis revealed low Cx43 protein levels in the lungs 0 h, 1 h, 8 h, 1 d, 2 d and 4 d after PBS challenge in the PBS/PBS group (Fig 4B). The Cx43 levels in OVA/OVA mouse lungs increased as time-dependent manner (Fig 4B, P <0.05, 0.01 or 0.001 for each time point compared with the following time points). And the Cx43 protein levels in OVA/OVA mice lung were significant higher than those of PBS/PBS group at the 1 h, 8 h, 1 d, 2 d and 4 d time points after the first challenge (P < 0.01 or 0.001).

### Localization of Cx43 in OVA/OVA mouse lung

Previous reports have shown that Cx43 plays an important role in inflammation and pathogenesis in many lung diseases [11,18,22,23]. We investigated the expression of the Cx43 protein in the lung at different time points after OVA challenge using immunofluorescence.

**Fig 5:**
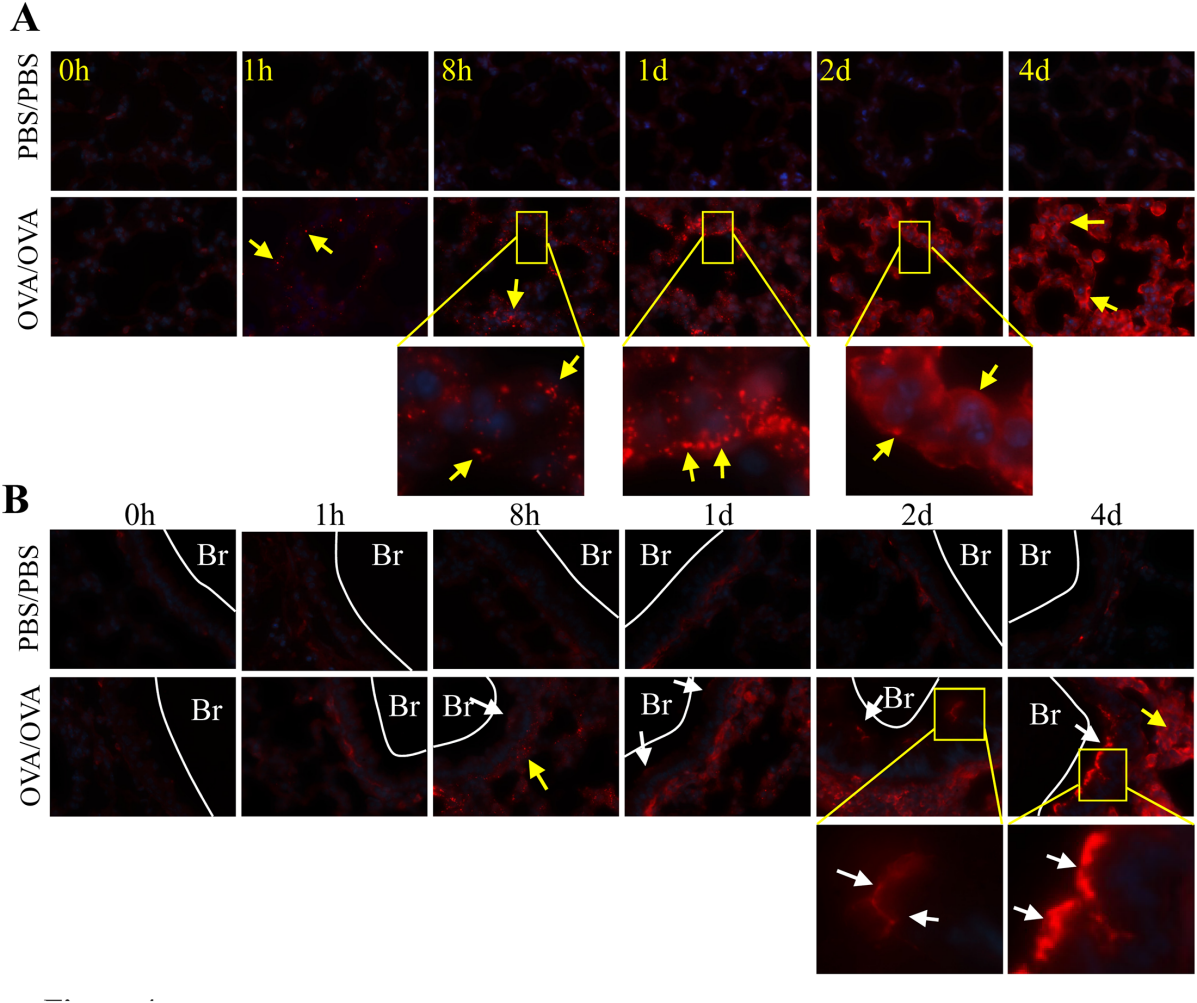
OVA-mediated Cx43 protein locates at both bronchial and alveolar cells of the lung. Samples were collected at 0 h, 1 h, 8 h, 1 d, 2 d and 4 d after the first challenge. (A) Cx43 expression in the pulmonary alveoli (yellow arrows) gradually increased from 1 h after the first challenge in the OVA/OVA group, but not in the PBS/PBS group. (B) Cx43 expression in the bronchial epithelium (white arrows) and in the surrounding pulmonary alveoli (yellow arrows) gradually rose from 8 h after the first challenge in the OVA/OVA group. The original magnification was ×1000. Br, bronchial; N = 6 in each group. Results are representative of three independent experiments.

There were no positive staining of Cx43 for negative controls. There was no detectible Cx43 in the PBS/PBS mice lung using immunofluorescence (Fig 5A, 0 h-4 d). However, OVA-mediated Cx43 induction (first as dots and then as sheets of immunofluorescent staining) was observed in the pulmonary alveoli and bronchia of the OVA/OVA group (Fig 5A and 5B). The OVA-mediated Cx43 induction appeared at 1 h and increased in a time-dependent manner in the pulmonary alveoli (Fig 5A). A similar pattern was observed in the bronchi and surrounding tissues in OVA/OVA mice (Fig 5B).

### The development of inflammatory infiltration and cytokine production during asthma induction

To investigate the development of inflammation during asthma progression, we evaluated lung inflammation and IL-4, IL-5, and IL-13 production in the BALF and OVA-specific IgE in the serum at different time points similar to those described above for Cx43 expression.

**Fig 6:**
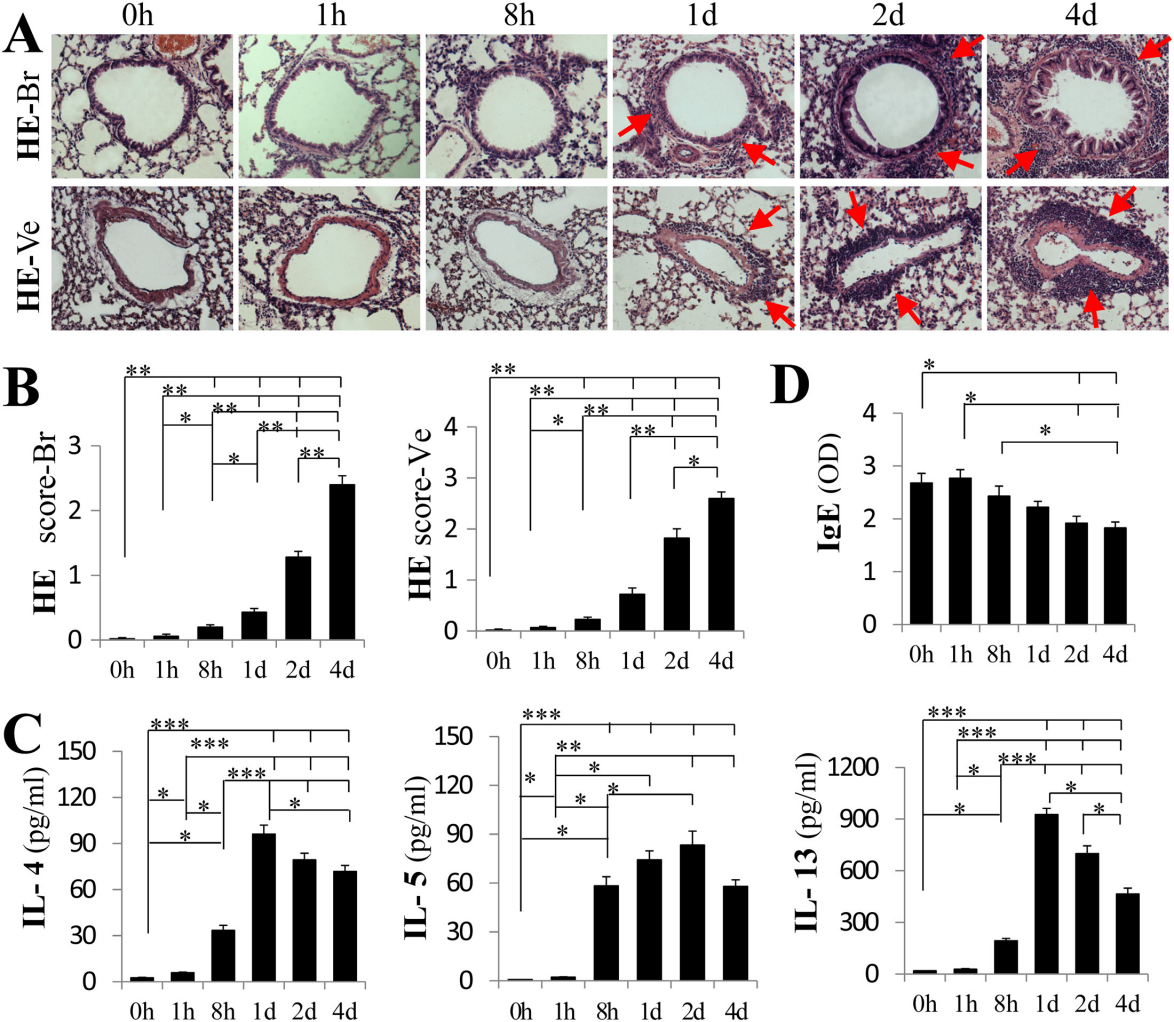
The temporal characteristics of inflammatory cell infiltration and cytokineon OVA-induced mouse lung. The samples were collected 0, 1 h, 8 h, 1 d, 2 d and 4 d after OVA challenge. (A) The inflammatory infiltrates (arrows) in the peribronchial (HE-Br) and perivessel (HE-Ve) tissues gradually increased in a time-dependent manner. The airway inflammation scores were based on HE staining of the peribronchial (HE-Br) and perivessel (HE-Ve) tissues (B). The IL-4, IL-5, and IL-13 levels were detected in the BALF (C) and OVA-specific IgE (D) was detected in the serum. The original magnification was × 200. Mean ± SEM. *, P < .05; **, P < .01; ***, P < .001. N = 6 in each group. Results are representative of two independent experiments.

No inflammatory cell infiltration was found 0 and 1 h after challenge. Inflammatory cells started to infiltrate around the bronchi and vessels at 8 h and reached to a maximum at day 4 (Fig 6A and 6B, P < 0.05 or 0.01 for the peribronchial and perivessel cells). IL-4, IL-5 and IL-13 levels in BALF also increased 8 h after the first challenge and reached their maximum values on day 1, day 2, and day 1, respectively (Fig 6C, P < 0.05 or 0.01 or 0.001). Serum OVA-specific IgE reached the highest level just before the first challenge (0 h) and then decreased but still remained high level until 4 days after the first challenge (Fig 6D, P < 0.05).

### Correlation between Cx43 expression levels and inflammatory parameters during asthma induction

**Fig 7:**
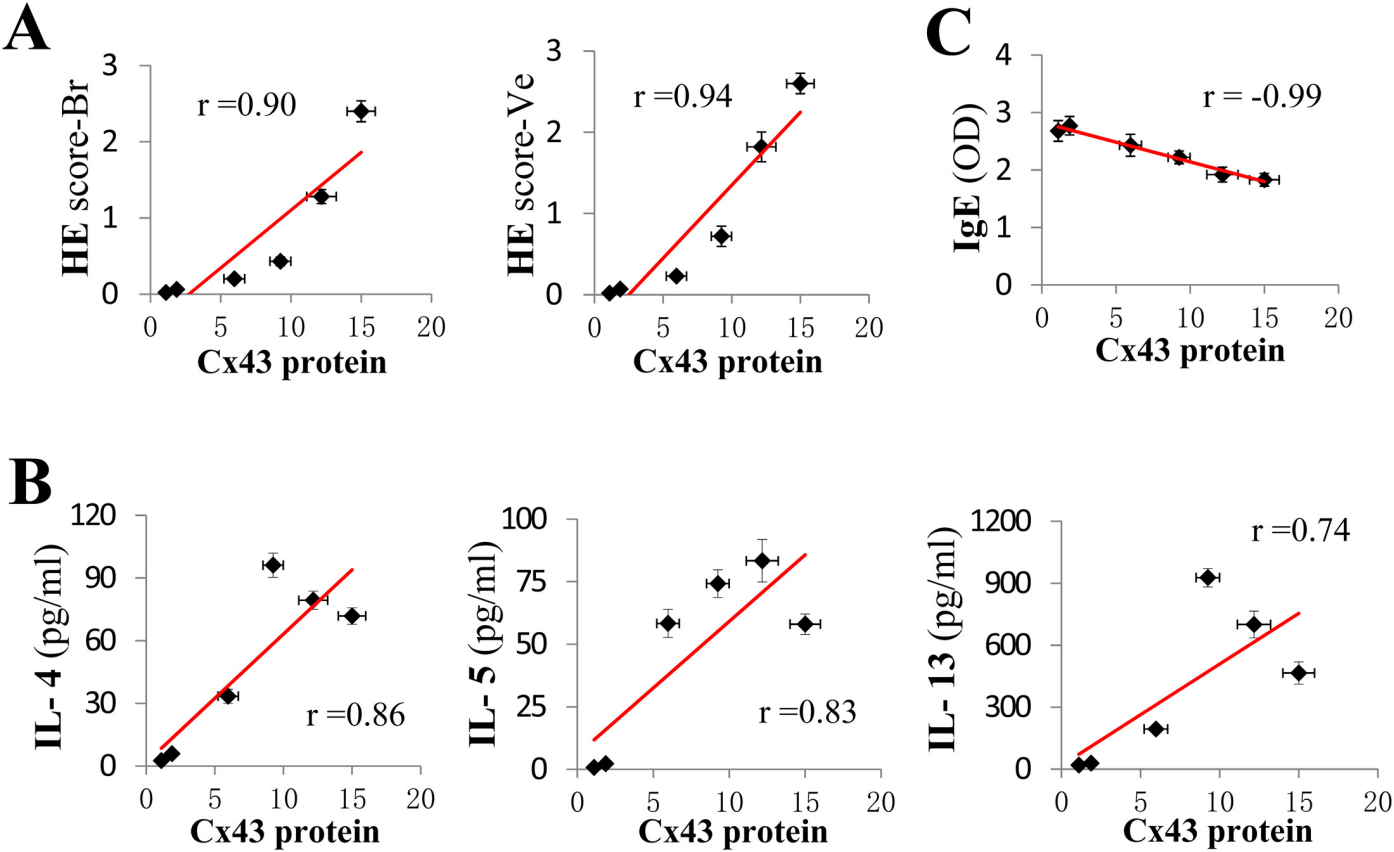
The correlation between the Cx43 levels and lung inflammation, interleukin and IgE levels. (A) The correlation between Cx43 protein levels and the inflammatory HE-Br and HE-Ve scores. The correlation between Cx43 protein levels and the IL-4, IL-5, and IL-13 levels in the BALF (B), and OVA-specific IgE levels in the serum (C). The dots represent the average HE scores or cytokine and IgE levels (Y axis), and Cx43 protein levels detected by western blot (X axis). Cx43 protein levels were analyzed at each time point after the first challenge relative to the 0 h time point in the control group (n = 6 in each group). Results are representative of two independent experiments.

To determine the role of Cx43 in OVA-challenge mediated inflammatory response, we analyzed the correlation of Cx43 protein level with lung inflammatory indices. There was a positive correlation between the Cx43 protein levels and the peribronchial and perivascular HE scores in OVA/OVA mouse lung (Fig 7A, P < 0.05, r = 0.903 and 0.942, respectively). There was also a positive correlation between Cx43 expression and IL-4 and IL-5, but not IL-13, in the BALF (Fig 7B, P < 0.05). Moreover, there was a negative correlation between Cx43 expression and the serum IgE levels (Fig 7C, P < 0.05).

## Discussion

In this study, three important findings have been characterized. First, OVA induces upregulation of Cx43 mRNA and protein in murine asthma. Second, Cx43 upregulation by OVA induction occurs at both bronchial and alveolar epithelial cells. Finally, Cx43 up-regulation is followed by structural and functional changes at the levels of the bronchi and blood vessel in OVA/OVA mice.

GJCs are intercellular channels that connect the cytoplasm of adjacent cells; they are formed by head-to-head docking of connexins [24,25]. GJCs allow the intercellular passage of small soluble molecules and even mitochondria between the same or different cell types [14,15,26]. The physiological role of GJCs in several tissues has been elucidated to be involved in connexins [15]. Mesenchymal stem cells were reported to protect pulmonary alveoli through Cx43-dependent cellular attachment [16]. As the most widely and highly expressed gap junction protein, Cx43 is ubiquitously expressed in almost all organs [27]. Alveolar epithelial and endothelial cells are the predominant cell types that express Cx43 in the lung [24,28,29]. Increasing evidence suggests that Cx43 is an inflammatory mediator that plays an important role in the pathogenesis of lung diseases such as ALI, CF and PAH [11]. Cx43-mediated calcium signaling in lung tissue was proposed to be important for the pathophysiology of acute inflammatory responses in the lungs [30,31]. Moreover, a recent report demonstrated that Cx43 contributes to neutrophil recruitment during acute lung inflammation induced by lipopolysaccharides in mice [18]. Cx43 has been reported to exhibit different characteristics in other diseases of respiratory system. For example, Cx43 was shown to be poorly expressed in human non-small cell lung cancer samples [32]. Decreased Cx43 expression was correlated with eosinophil infiltration in nasal polyps [33].

Previous studies showed that blocking or inhibiting the function of Cx43 could attenuate some diseases in animals. Silencing of Cx43 suppressed the invasion, migration and lung metastasis of rat hepatocellular carcinoma cells [34]. Intratracheal instillation of a specific Cx43 inhibitor efficiently reduced neutrophil recruitment from the blood circulation into the lungs in an acute lung injury mouse model [18]. However the importance of Cx43 in OVA-induced allergic airway inflammation has not been thoroughly studied.

In current study, we found that immunoreactive Cx43 was mostly localized in the alveolar and bronchial epithelial layers in asthmatic lungs. There was a gradual increase in Cx43 mRNA and protein expression levels after OVA inhalation. In addition, OVA mediated Cx43 induction occured earlier than other inflammatory indices, such as the inflammatory cell infiltration and the increase of IL-4, IL-5 and IL-13 etc. Moreover, Cx43 protein levels significantly and positively correlate with airway inflammatory cell infiltration and IL-4 and IL-5 production in the BALF, respectively. These results indicate that Cx43 may play a regulatory role in asthma inflammatory response. Furthermore, these results suggest that Cx43 may be a target for the treatment of the asthmatic inflammation.

In conclusion, Cx43 protein levels showed a significant positive correlation with airway inflammation, and the initiation of the Cx43 increase occurred prior to inflammatory cell infiltration. This finding indicates that Cx43 is a potential therapeutic target for asthma.
